# Vestibular and visual influence on postural stability and egomotion perception in persistent postural-perceptual dizziness (PPPD)

**DOI:** 10.1007/s00415-026-13653-z

**Published:** 2026-02-09

**Authors:** Renana Storm, Skadi Gerkensmeier, Hannah Keller, Pia Herborn, Andreas Sprenger, Christoph Helmchen

**Affiliations:** 1 Department of Neurology, University Hospital Schleswig-Holstein, Campus Lübeck, Ratzeburger Allee 160, 23538 Lübeck, Germany; 2 Center of Brain, Behavior and Metabolism (CBBM), University of Lübeck, Marie-Curie-Straße, 23562 Lübeck, Germany; 3 Institute of Psychology, University of Lübeck, Lübeck, Germany

**Keywords:** PPPD, Postural, Visual, GVS

## Abstract

**Background:**

Patients suffering from persistent postural-perceptual dizziness (PPPD) often experience postural instability that worsens when exposed to visual motion stimuli. We investigated how different visual motion stimuli affect patients’ postural sway and their perceived egomotion during stance.

**Methods:**

28 PPPD patients and 26 gender and healthy control subjects (HC) underwent posturographic measurements on a firm or foam platform while being exposed to either vestibular or visual motion stimuli or their combination. Vestibular stimuli were applied via 1.3 mA galvanic vestibular stimulation (GVS) or a *sham* stimulus. Visual stimulation (VS) was performed via 20-s video snippets of a silent movie, flow-field animation, or a rollercoaster video from the driver’s perspective. Outcome measures included postural sway speed (PSS) and perceived egomotion, collected via self-ratings after each trial.

**Results:**

Compared to HC, PSS of PPPD patients was higher on a firm surface during vestibular stimulation alone and combined visual–vestibular stimulation (except during rollercoaster VS) but not during VS alone. These group differences disappeared on foam, except during the baseline (noVS, noGVS) condition. Egomotion perception was rated consistently higher by PPPD participants in all conditions but in a non-linear ratio.

**Conclusion:**

Our visual motion stimuli were capable of eliciting different magnitudes of perceived egomotion and postural sway without significant group differences in postural sway challenging the notion of increased visual sensitivity in PPPD. Multisensory stimulation alleviates visual sensitivity and counteracts postural misperception in quiet stance. Patients’ non-linear increase of egomotion with increasing postural sway differs from HC and reflects a non-linear perceptual-postural scaling as a crucial mechanism in PPPD.

**Supplementary Information:**

The online version contains supplementary material available at 10.1007/s00415-026-13653-z.

## Introduction

Many neurological disorders affecting cerebellar, vestibular (vestibulopathy), and proprioceptive signal processing (polyneuropathy) elicit perceived unsteadiness. Patients with persistent postural-perceptual dizziness (PPPD), one of the most frequent chronic dizziness disorders [25], also complain about dizziness and abnormal egomotion perception, often in the absence of objective unsteadiness, sensory or cerebellar abnormalities. Many of them had experienced and recovered from a previous vestibular disorder/disease (e.g., benign paroxysmal positional vertigo, vestibulopathy), but the nature of this postural misperception remains unclear.

PPPD is classified as a functional neurological disorder that is associated with an increased vigilance for the body and environment [25]. One of the diagnostic criteria of PPPD is the exacerbation of symptoms by upright posture, exposure to moving visual stimuli or complex visual patterns as well as active or passive movements, i.e., vestibular stimulation [26]. Usually, one of these exacerbation factors is more prominent in patients than the others [1]. Accordingly, four PPPD subtypes have been proposed: visual intolerance, intolerance to quiet standing or sitting, active motion intolerance, and passive motion intolerance [1]. The active motion intolerance subtype appears to be the most prominent subtype followed by visual and quiet standing intolerance, irrespective of a previous vestibular disease.

The mechanisms underlying sensory dependence of unsteadiness in PPPD, however, remain undetermined. They may result from abnormal sensory-perceptual or multi-sensory (visual, vestibular, somatosensory) processing, leading to abnormal motion detection and egomotion perception. PPPD patients seem to rely on stationary as well as visual motion cues in their balance control [17, 21, 23]. Larger brain excitability in response to sensory (visual, vestibular) stimulation has been reported [21, 28] as well as altered sensory perceptional thresholds of motion detection [27]. Visual motion detection thresholds are higher (poorer) in PPPD, provoking the risk of later detection of moving visual stimuli. In contrast, egomotion perception thresholds by galvanic vestibular stimulation (GVS) are lower compared to healthy control subjects (HC), making PPPD patients prone to recognize egomotion even in non-motion conditions [27, 30].

Postural control of PPPD patients was poorer compared to HC during a sensory organization test (SOT) when subjects were exposed to different somatosensory, vestibular, and visual signals. This was even found when HC were examined under more challenging conditions, with eyes closed or exposure to moving visual surroundings [17, 23]. This stands in contrast to other studies demonstrating abnormal postural sway in PPPD patients particularly in simple ‘baseline condition’, e.g., standing on a firm platform with the eyes open without visual motion information. Importantly, the abnormal postural sway normalizes (becomes indistinguishable from HC’s) in more demanding tasks, i.e., standing on foam with eyes closed [9, 23, 30]. This argues against an abnormal multi-sensory integration causing patients’ unsteadiness and rather reflects a sensory-perceptual amplification [28]. However, this has not yet been systematically studied using moving visual stimuli or combined sensory (visual and vestibular) stimuli that evoke egomotion perception. Patients’ postural sway became higher compared to that of HC during visual fixation when facing rotating dots, but this visual sensitivity was not different from that of a group of dizzy non-PPPD patients [4]. Evoked brain activity in the visual cortex (V1–V3) of PPPD patients’ in response to visual virtual reality rollercoaster simulation was larger compared to that of HC and increased with the dizziness handicap inventory [21]. Unfortunately, this increased sensitivity of the visual cortex has neither been related to postural imbalance nor to egomotion perception yet. When exposed to galvanic vestibular stimuli evoking egomotion perception, patients’ postural sway was higher compared to HC [9, 30].

Importantly, PPPD patients perceive their unsteadiness and egomotion in a quiet stance on a solid ground higher compared to their quantitatively recorded sway. The ratio of perceived egomotion to observed (recorded) postural sway is disproportionately high compared to both HC and patients with vestibulopathy. This has lead to the concept of “postural misperception” in PPPD [18]. This misperception seems to reverse with more demanding tasks [9, 18], but the impact of sensory motion cues during these tasks on the perceptional improvement is unknown. When asked to reproduce their perceived sway, patients exhibit a much higher postural sway compared to the recorded sway. This excessive reproduced sway suggests an abnormal postural-perceptual scaling which can be reversed by (galvanic) vestibular stimulation [9].

Under real-world circumstances, PPPD patients are often exposed to combined moving visual and vestibular stimuli. Accordingly, we investigated how the combination of visual and vestibular stimulation evoking various levels of egomotion affects both postural sway and immediate perceived egomotion. We compared the effect of visual, vestibular, or combined stimulation on postural sway and perceived egomotion in PPPD patients and HC while they were exposed to different degrees of sensory provoked egomotion. We hypothesized that (i) the difference in postural sway between PPPD and HC is higher during baseline conditions than during more demanding tasks, (ii) egomotion perception is higher in patients than in HC for all vestibular and visual stimuli, (iii) vestibular stimulation has a greater impact compared to visual stimulation on both postural sway and egomotion perception in PPPD patients and HC, (iv) additional vestibular stimulation rather decreases visual sensitivity of postural sway in PPPD patients, and (v) PPPD patients with the visual and quiet standing subtypes experience a higher level of egomotion compared to the active motion intolerance subtype.

## Methods

### Participants

28 (10 male) PPPD patients (17 with primary PPPD) and 26 (11 male) age-matched healthy control subjects (HC) participated in this study. PPPD patients were diagnosed at the Centre for Vertigo and Balance Disorders (University of Luebeck/Germany) according to the disease criteria by the Bárány Society [26]. We calculated a cohort size using a power analysis revealing 26 participants per group (G*Power 3.1.9.7 [5]; effect size 0.8, alpha probability of 0.05, power of 0.8). All participants underwent a neurologic and neuro-otologic examination as part of the study (for details see: Testing of vestibular function). All of them had normal visual acuity and showed normal vestibular function on quantitative testing [head impulse test, vestibular-evoked myogenic potentials, subjective visual vertical (SVV)]. HC were recruited via flyers on- and off-campus and screened for past and current symptoms of vertigo, dizziness, migraine, or other types of neurological and balance disorders prior to the experiment. Exclusion criteria included cerebral lesions, psychiatric disorders (i.e., depression, anxiety disorders), polyneuropathy, or intake of sedating medication. Symptom severity of PPPD was assessed using the Niigata PPPD Questionnaire (NPQ) [31] and the Athens–Lübeck Questionnaire (ALQ) [1]. For characterization, we used the Motion Sickness Susceptibility Questionnaire (MSSQ) [8], the anxiety and depression scores of the Hospital Anxiety and Depression Scale (HADS) [11, 19], the neuroticism and extraversion scores of the NEO-Five Factor Inventory (NEO-FFI), and the Visual Analog Values of the EQ-5D-3L [12] (see Table 1). In short, PPPD patients revealed higher values of symptom exacerbation (NPQ, ALQ), for neuroticism (NEO-FFI), the anxiety and depression scale (HADS) and the self-assessment for daily life quality (EQ-5D-3L). Patient revealed lower scores for extraversion (NEO-FFI). The study protocol was approved by the local Ethics Committee of the University of Luebeck (AZ 17-036, AZ 21-098). Written informed consent was obtained from all participants.

**Table 1:** Demographics and clinical scores of participants

	PPPD (mean ± SD)	HC (mean ± SD)	Statistical significance (*p*)
Number	28	26	n.s.
Disease duration (months)	34.86 ± 40.47	N/A	N/A
ALQ total	17.79 ± 6.37	0.73 ± 1.82	< 0.001
ALQstand	4.50 ± 2.38	0.12 ± 0.43	< 0.001
ALQvis	4.93 ± 1.94	0.08 ± 0.39	< 0.001
ALQpass	3.39 ± 2.20	0.38 ± 0.85	< 0.001
ALQact	4.96 ± 1.77	0.15 ± 0.46	< 0.001
Niigata total	32.25 ± 13.74	1.27 ± 2.75	< 0.001
Upright posture	11.29 ± 5.71	0.19 ± 0.80	< 0.001
Movement	11.29 ± 4.33	0.65 ± 1.23	< 0.001
Visual stimulation	9.68 ± 6.01	0.42 ± 1.24	< 0.001
HADS-D	7.18 ± 3.72	1.38 ± 2.25	< 0.001
HADS-A	8.93 ± 3.78	3.19 ± 2.76	< 0.001
MSSQ	12.48 ± 10.42	8.10 ± 7.80	0.085
NEO-FFI (Neuroticism)	24.14 ± 6.22	14.78 ± 7.43	< 0.001
NEO-FFI (Extraversion)	23.64 ± 6.69	29.41 ± 8.06	0.006
EQ-VAS	61.40 ± 19.18	90.38 ± 6.50	< 0.001

*ALQ* Athens–Lübeck Questionnaire, *ALQstand* intolerance to quiet standing, *ALQvis* visual intolerance, *ALQpass* passive motion intolerance, *ALQact* active motion intolerance, *EQ-VAS* visual analog values of the EQ-5D-3L, *HADS* Hospital Anxiety and Depression Scale, *HC* healthy control subjects, *MSSQ* Motion Sickness Susceptibility Questionnaire, *n/a* not applicable, *NEO-FFI* NEO-Five Factor Inventory, *n.s.* not significant, *PPPD* persistent postural-perceptual dizziness.

### Testing of vestibular function

Vestibular function was assessed using quantitative head impulse testing (qHIT), vestibular-evoked myogenic potential testing, and quantification of the subjective visual vertical. qHIT was assessed using the EyeSeeCam® HIT System (Autronics, Hamburg, Germany) at a sampling rate of 220 Hz. Quantitative HIT was delivered by passive head impulses (HIT) with rapid small amplitude (10°–15°) horizontal head rotations (3000°–4500°/s) while the participant was sitting on a chair fixating a red LED at a distance of 100 cm. Vestibular–ocular reflex (VOR) gain > 0.7 was defined as unremarkable. For further details see [14, 15]. The subjective visual vertical was assessed with the head fixed on a chin rest by the subject’s adjustment of a bar to the perceived visual vertical without any spatial orientation clues in a dotted half-spherical dome, which is stationary or dynamic (moving visual background) around the line of sight. The normal range of SVV was defined as deviation of <  ± 2.5°.

### Galvanic vestibular stimulation (GVS) and visual stimulation (VS)

Bilateral galvanic vestibular stimulation (GVS) was applied on the mastoids using a bipolar constant current stimulator (DS5 model, Digitimer Ltd., U.K.) with skin contact electrodes provided by EasyCap GmbH (Herrsching/Germany). Galvanic stimulation was delivered using a low-frequency alternating current which passed between the two mastoid electrodes. The stimulation site was pre-treated with local anesthetics (Anesderm® lotion, Pierre Fabre Dermo-Kosmetik GmbH, Freiburg/Germany) 30 min prior the experiment to minimize potential nociceptive stimulation. Each stimulation lasted 20 s with 1 Hz alternation. All subjects indicated a medio-lateral motion direction. Depending on the condition, they received either (i) no stimulation (*no*GVS), (ii) galvanic vestibular stimulation (GVS): 1.3 mA with 100 ms linear onset and offset ramps, followed by a 300 ms stimulation plateau or (iii) sham stimulation (*sham*GVS): 1.3 mA (GVS) with 100 ms linear onset followed by 400 ms without stimulation that usually does not elicit directional to and fro sway perception, but participants may notice some weak tingling.

GVS conditions were paired with three types of visual stimulation (VS), designed to differ by the degree of elicited egomotion: a black-and-white silent movie (movie), a flow-field animation (flow-field), and a rollercoaster video from the driver’s perspective (rollercoaster). For movie VS, sections of the movie “The Artist” (Hazanavicius, 2011) were trimmed to 20-s snippets. Flowfield stimulation was programmed using Matlab (version R2022b, The MathWorks, Natick/MA) and Psychophysics Toolbox 3 extension (version 3.0.16, [3], see supplementary information for a video example [FlowField.mp4]). For the rollercoaster VS, sections of publicly available point-of-view rollercoaster videos (see *Video References* in the supplementary information) were trimmed to 20-s snippets. VS were presented on a height-adjustable, wall-mounted, curved (1500R) monitor (G32CQ4DE E2, 120 Hz, resolution 2560 × 1440 Pixel), in approximately 60 cm distance to the viewer’s eyes. During trials where only GVS was applied, a centered red dot (size: 0.5°) was presented on the monitor. After each trial, participants rated their perceived egomotion on a scale from 0 (“no egomotion”) to 100 (“felt as if falling off the platform”). Ratings were provided via joystick (Logitech Extreme 3D pro Precision, Lausanne, Schweiz), using a triangle-shaped visual analog scale to facilitate intuitive rating.

### Posturography

We used a Kistler force platform (Model 9260AA6, Kistler Instrumente AG, Winterthur, Switzerland; 50 cm width, 60 cm length) equipped with piezo-electric 3-component force sensors for recording postural changes during the above-mentioned experimental conditions in a similar way as described elsewhere [9, 29, 30]. The displacement of the center of pressure (CoP) in the medial–lateral (ML) and the anterior–posterior (AP) directions were recorded and the sum vector calculated using Matlab® (R2024b, The Mathworks, Natick/MA). Results are given as the median postural sway speed (PSS, in mm/s), calculated from the AP and ML movements. Postural sway was recorded in intervals of 20 s for off-line analysis (sampling frequency 250 Hz) [10, 24]. PSS has been shown as a robust, discriminative, and reliable factor of recording postural balance [7, 20]. Participants were instructed to stand upright, with their feet parallel and arms hanging next to the body during the recordings. For the firm platform condition, they stood on top of a wooden board. For the foam platform condition, a foam rubber pad (50 × 60 × 10 cm, compression hardness: 3.3 kPa, volumetric weight: 40 kg/m^3^) was placed underneath the wooden board. All participants weighed between 60 and 120 kg to ensure a correct use of the foam rubber pad.

### Experimental setup

**Fig. 1:**
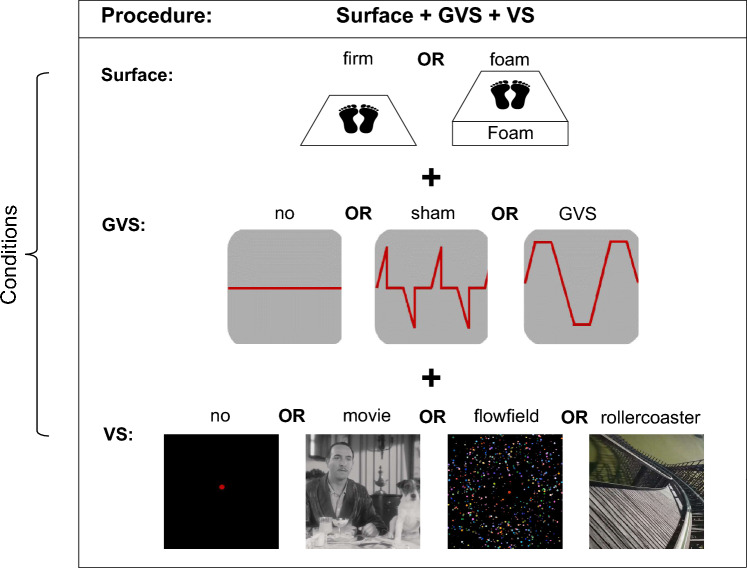
Experimental setup. Each trial was run by the same procedure (surface + GVS + VS) which included the conditions listed below. Each condition was combined with each other, i.e., firm surface + *no*GVS + no VS, resulting in a 2 × 3 × 4 factorial design (surface × GVS × VS). *GVS* galvanic vestibular stimulation, *VS* vestibular stimulation

After ensuring a sufficient application time of the local anesthetic (at least 30 min), the GVS electrodes were placed on the mastoids bilaterally, and *sham*GVS and GVS stimulus were presented. Subsequently, participants stood on the Kistler force platform for posturographic recordings with the monitor 60 cm in front of them and the joystick on their right-hand side to rate their level of egomotion perception after each condition. During each condition, the investigator stood behind the participant to prevent them from falling if necessary. Each participant underwent the same experimental setup due to technical reasons while VS videos were randomized. Each condition started on the firm platform with eyes open, followed by visual and vestibular stimulation, separately and combined (see Fig. 1). All conditions were then repeated on the foam rubber pad. For better lighting conditions, the room light was semi-dimmed.

### Statistical analysis

Statistical analysis was conducted in R (version 4.4.3) using the RStudio interface (version 2024.12.1+563). The following packages were used (versions in parentheses): afex (1.4-1), emmeans (1.10.7), tidyverse (2.0.0), dplyr (1.1.4), ggplot2 (3.5.1), lme4 (1.1.38) [2], and ggpubr (0.6.0). All analyses were initially conducted separately for patients with primary and secondary PPPD. However, as no significant differences in sway speed or egomotion ratings were observed between the groups (*p* always > 0.172 for all comparisons), data were subsequently pooled for all further analyses. To complement the ANOVA-based analysis, data were additionally analyzed using linear mixed-effects models estimated via restricted maximum likelihood (REML) [16]. This allows variance decomposition into visual- and vestibular-related components of postural sway speed and egomotion perception (see Supplementary Table S9 and S10).

The experimental design included three within-subject factors: SURFACE (Firm, Foam), GVS (*no*, *sham*, GVS), and VS (no, movie, flow-field, and rollercoaster), as well as the between-subject factor GROUP (HC, PPPD). For facilitated interpretability of the results, analysis was done separately for firm and foam conditions. PSS and egomotion perception (rating) were compared using a separate Analysis of Variance (ANOVA). For correlation analysis with questionnaires and disease parameters, Spearman rho nonparametric correlation was used. The relationship between increase in individual egomotion perception and PSS was visualized using a logarithmic fit (a*log(*x*) + *b*). Egomotion perception values were normalized to a maximum rating of 1 (corresponding to 100%), and PSS values were normalized to a maximum of 100 mm/s, representing the 95th percentile of PSS. The area under the fitted curve (AUC) was subsequently compared between PPPD and HC.

Statistical significance was defined as *p* < 0.05. Data reported indicate mean values (*M*) and standard deviation (SD) unless otherwise stated. Post hoc comparisons were Bonferroni-corrected for multiple testing. Results are visualized in boxplots, with lower and upper hinges representing the 25th and 75th percentiles and whiskers extending to 1.5 times the interquartile range.

## Results

Results are systematically presented for each condition [(i) vestibular GVS > (ii) visual and > (iii) combined visual-vestibular stimulation] in the same order (effects on postural sway followed by egomotion perception) starting with main effects, interactions, and subsequent post hoc comparisons.

### Galvanic vestibular stimulation (GVS)

**Fig. 2:**
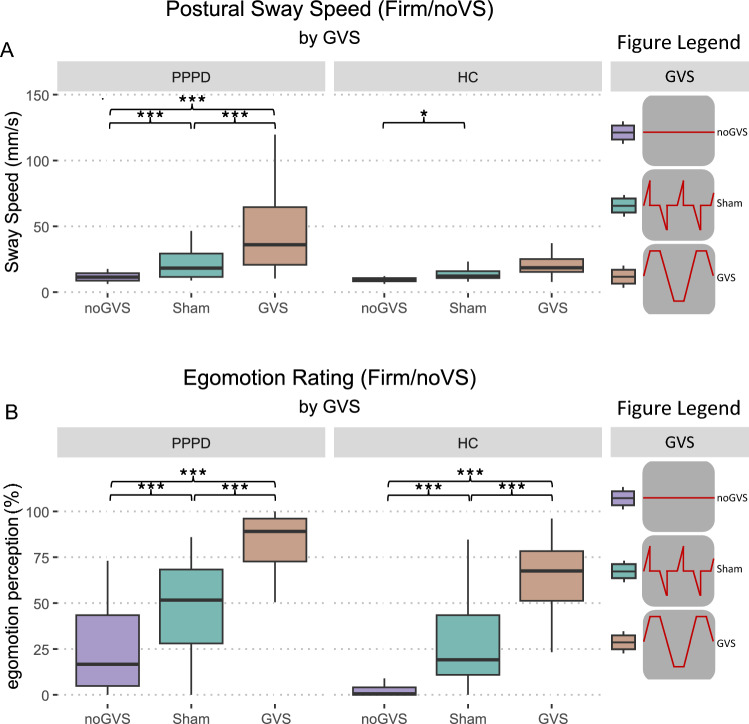
Vestibular stimulation (GVS). PSS (**A**) and egomotion perception (**B**) during *no*, *sham* and GVS without VS for PPPD patients and HC on firm surface. GVS elicits the highest level of PSS and egomotion perception group-independent. However, patients’ exact values are elevated when compared to HC, even in baseline conditions (*no*GVS). * ≤ 0.05, *** < 0.001. *GVS* galvanic vestibular stimulation, *HC* healthy control subjects, *PPPD* persistent postural-perceptual dizziness, *PSS* postural sway speed, *VS* visual stimulation

For the first analysis, we looked at the effect of GVS (*no*, *sham*, GVS) without VS on PSS, while participants stood on a firm platform. There was a main effect for GROUP (*F*(1, 52) = 16.74, *p* < 0.001, *η*^2^ = 0.127), GVS (*F*(2,104) = 36.27, *p* < 0.001, *η*^2^ = 0.277) and an interaction between GROUP × GVS (*F*(2, 104) = 11.24, *p* < 0.001, *η*^2^ = 0.106). Post hoc comparisons between PPPD patients and HC showed an increased PSS for patients under *no*GVS (*t*(52) = 2.68, *p* = 0.01, *Δ* = 2.6), *sham* (*t*(52) = 2.85, *p* = 0.006, *Δ* = 6.84), and GVS (*t*(52) = 3.79, *p* < 0.001, *Δ* = 29.02). Within-group differences between GVS conditions are marked in Fig. 2A, exact values can be found in the supplementary information (see Supplementary Table S1).

Regarding the egomotion perception, there was a main effect for GROUP (*F*(1, 52) = 22.0, *p* < 0.001, *η*^2^ = 0.169) and GVS (*F*(2, 104) = 154.57, *p* < 0.001, *η*^2^ = 0.606) but no interaction between GROUP × GVS (*p* = 0.82). Post hoc comparisons revealed higher perceived egomotion in PPPD patients compared to HC for all GVS conditions (*no*GVS: *t*(52) = 3.3, *p* = 0.002, *Δ* = 16.49; *sham*GVS: *t*(52) = 3.12, *p* = 0.003, *Δ* = 20.05, GVS: *t*(52) = 3.12, *p* = 0.003, *Δ* = 20.05). Within-group differences between GVS conditions are marked in Fig. 2B, exact values can be found in the supplementary information (see Supplementary Table S2).

### Visual stimulation (VS)

**Fig. 3:**
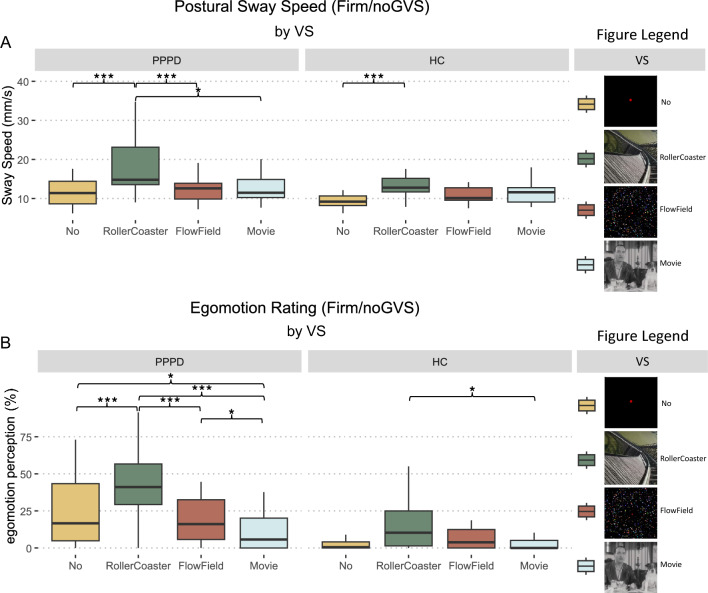
Visual stimulation (VS). PSS (**A**) and egomotion perception (**B**) for no, movie, flow-field and rollercoaster VS without GVS in PPPD patients and HC on firm surface. Patients showed an increased PSS during no VS compared to HC but not during any VS condition. Rollercoaster VS induced the highest level of PSS in both patients and HC. Egomotion perception (**B**) was increased in patients for all VS conditions compared to HC. * ≤ 0.05, *** < 0.001. *HC* healthy control subjects, *PPPD* persistent postural-perceptual dizziness, *PSS* postural sway speed, *VS* visual stimulation

Investigating the effect of VS (no, movie, flow-field, rollercoaster) in the absence of GVS on PSS on the firm platform, there was a main effect for VS (*F*(3, 156) = 17.23, *p* < 0.001, *η*^2^ = 0.127) a trend for GROUP (*F*(1, 52) = 3.02, *p* = 0.088, *η*^2^ = 0.032) but no interaction for GROUP × VS (*p* = 0.585). Post hoc comparisons showed a higher PSS in patients during no VS (*t*(52) = 2.68, *p* = 0.01, *Δ* = 2.68), with comparable PSS for the remaining conditions (movie: *p* = 0.16, flow-field: *p* = 0.69, rollercoaster: *p* = 0.16). Within-group differences between VS conditions are marked in Fig. 3A, exact values can be found in the supplementary information (see Supplementary Table S3).

Regarding egomotion perception, there was a main effect for VS (*F*(3, 156) = 21.61, *p* < 0.001, *η*^2^ = 0.183), for GROUP (*F*(1, 52) = 24.57, *p* < 0.001, *η*^2^ = 0.179) and a significant interaction between GROUP × VS (*F*(3, 156) = 3.71, *p* = 0.013, *η*^2^ = 0.037). Post hoc comparisons between PPPD and HC showed that PPPD rated their perceived egomotion higher for each VS condition: no VS (*t*(52) = 3.30, *p* = 0.002, *Δ* = 16.5), movie (*t*(52) = 2.30, *p* = 0.025, *Δ* = 5.57), flow-field (*t*(52) = 4.0, *p* < 0.001, *Δ* = 12.66), rollercoaster (*t*(52) = 4.05, *p* < 0.001, *Δ* = 25.4). Within-group differences between VS conditions are marked in Fig. 3B, exact values can be found in the supplementary information (see Supplementary Table S4).

### Galvanic vestibular stimulation and visual stimulation (GVS + VS)

**Fig. 4:**
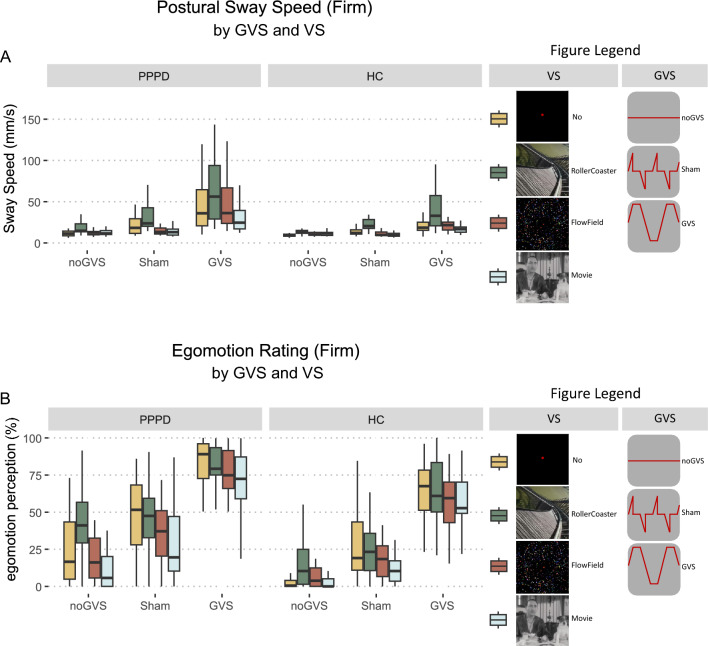
Combined stimulation (firm platform). PSS (**A**) and egomotion perception (**B**) during GVS (*no*, *sham*, GVS) and VS (no, movie, flow-field and rollercoaster) in PPPD patients and HC on firm surface. In general, GVS combined with rollercoaster VS elicited higher PSS group-independent with patients showing elevated exact values. Egomotion perception (**B**) was increased in patients during all GVS and VS combinations when compared to HC. With GVS, rollercoaster VS was no longer distinguishable between flow-field and no VS in PPPD patients, also seen in HC. *GVS* galvanic vestibular stimulation, *HC* healthy control subjects, *PPPD* persistent postural-perceptual dizziness, *PSS* postural sway speed, *VS* visual stimulation

Looking at the combined effect of both GVS (*no*, *sham*, GVS) and VS (no, movie, flow-field, rollercoaster) on PSS on the firm platform, there was a main effect for GROUP (*F*(1, 52) = 12.30, *p* = 0.001, *η*^2^ = 0.059), GVS (*F*(2, 104) = 77.25, *p* < 0.001, *η*^2^ = 0.235), and VS (*F*(3,156) = 4.45, *p* < 0.001, *η*^2^ = 0.115). The interaction between GROUP × GVS (*F*(2, 104) = 10.87, *p* < 0.001, *η*^2^ = 0.041) and GVS × VS (*F*(6, 312) = 10.11, *p* < 0.001, *η*^2^ = 0.047) was significant, but neither the interaction between GROUP × VS (*p* = 0.617) nor the three-way interaction GROUP × GVS × VS (*p* = 0.143) reached significance. Post hoc comparisons for every condition of GVS revealed an increased PSS in PPPD patients compared to HC for *sham*GVS (*t*(52) = 2.50, *p* = 0.016, *Δ* = 6.32) and GVS (*t*(52) = 3.61, *p* < 0.001, *Δ* = 20.89) which was not apparent without GVS (*no*GVS: *p* = 0.088, Fig. 4A). To portrait the main effect of GROUP in regard to VS, we calculated post hoc comparisons for every condition of VS. Patients showed higher PSS compared to HC for movie VS (*t*(52) = 3.31, *p* = 0.002, *Δ* = 7.05), flow-field (*t*(52) = 3.86, *p* < 0 0.001, *Δ* = 10.62) and no VS (*t*(52) = 4.09*, p* < 0.001, *Δ* = 12.82) but not for rollercoaster VS (*p* = 0.193). As we are specifically interested in the effect of complex visual stimulation (rollercoaster) and found a significant interaction between VS and GVS, we looked at the difference between rollercoaster and noVS under different conditions of GVS for both groups: PPPD patients showed an increased PSS during rollercoaster VS compared to no VS for *no*GVS (*t*(52) = 4.56, *p* < 0.001, *Δ* = 5.82) and *sham*GVS (*t*(52) = 3.14, *p* = 0.017, *Δ* = 15.13) but not for GVS (*p* = 0.44). In HC, PSS during rollercoaster compared to no VS was increased for *no*GVS (*t*(52) = 4.31, *p* < 0.001, *Δ* = 5.7) and GVS (*t*(52) = 3.7, *p* = 0.003, *Δ* = 29.7) but not for *sham*GVS (*p* = 0.08). All post hoc comparisons are listed in the supplementary information (see Supplementary Table S5).

Regarding egomotion perception, there was a main effect for GROUP (*F*(1, 52) = 30.01, *p* < 0.001, *η*^2^ = 0.173), GVS (*F*(2, 104) = 360.37, *p* < 0.001, *η*^2^ = 0.593) and VS (*F*(3, 156) = 25.89, *p* < 0.001, *η*^2^ = 0.092). Apart from the interaction between GVS × VS (*F*(6, 312) = 5.70, *p* < 0.001, *η*^2^ = 0.024), no interaction reached significance (*p* always > 0.158). As indicated by the main effects, but missing interactions between GROUP and both VS and GVS, PPPD patients rated their perceived egomotion higher than HC under all VS and GVS conditions (*p* always < 0.01, Fig. 4B). Evaluating the effect of complex VS (rollercoaster) on egomotion perception regarding the different GVS conditions, patients rated their egomotion perception during rollercoaster VS compared to no VS higher for *no*GVS (*t*(52) = 3.87, *p* = 0.002, *Δ* = 18.81) but not for *sham* or GVS (*p* > 1 for both). HC showed no significant differences in egomotion perception when comparing rollercoaster VS and no VS (*p* always > 1). All post hoc comparisons are listed in the supplementary information (see Supplementary Table S6).

**Fig. 5:**
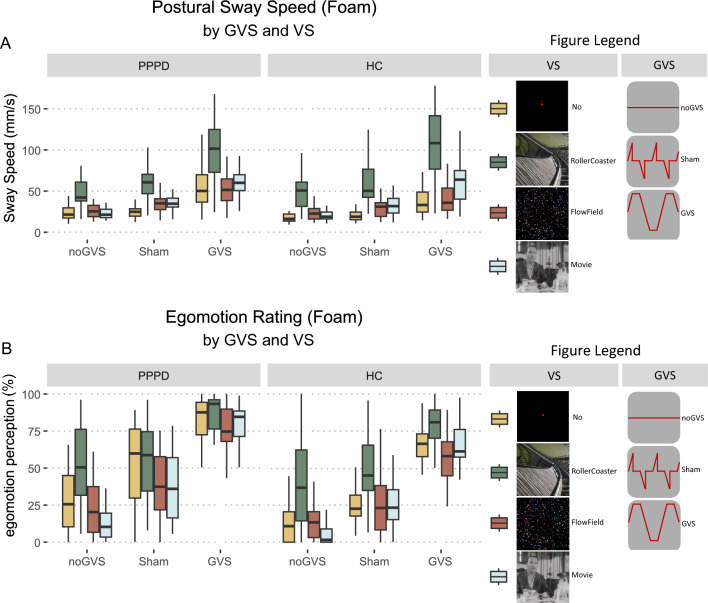
Combined stimulation (foam). PSS (**A**) and egomotion perception (**B**) during GVS (*no*, *sham*, GVS) and VS (no, movie, flow-field, and rollercoaster) in PPPD patients and HC on foam surface. PSS did not differ between groups except for the no VS condition. Rollercoaster VS triggered the highest PSS amplified by GVS. Egomotion perception (**B**) was higher in PPPD patients compared to HC independent of GVS but not independent of VS: rollercoaster VS was no longer different between groups. With GVS, no one of the VS was distinguishable in PPPD patients, whereas HC showed increased egomotion perception during rollercoaster VS, independent of GVS. *GVS* galvanic vestibular stimulation, *HC* healthy control subjects, *PPPD* persistent postural-perceptual dizziness, *PSS* postural sway speed, *VS* visual stimulation

Looking at the effect of GVS (*no*, *sham*, GVS) and VS (no, movie, flow-field, rollercoaster) on PSS under foam platform conditions, there was a main effect for GVS (*F*(2, 104) = 159.48, *p* < 0.001, *η*^2^ = 0.351) and VS (*F*(3, 156) = 127.91, *p* < 0.001, *η*^2^ = 0.357) but not for GROUP (*p* = 0.438). Significant interactions emerged between GROUP and VS (*F*(6, 312) = 22.75, *p* < 0.001, *η*^2^ = 0.054) as well as between GROUP × VS × GVS (*F*(6, 312) = 3.17, *p* = 0.005, *η*^2^ = 0.008) but not between GROUP × GVS (*p* = 0.924, Fig. 5A). Post hoc comparison for GVS showed no PSS differences between PPPD and HC (*p* always > 0.37). Post hoc comparison for VS revealed an increased sway speed in PPPD patients for no VS (*t*(52) = 2.86, *p* = 0.006, *Δ* = 9.47) which was not apparent in movie, flow-field or rollercoaster VS (*p* always > 0.17). For complex VS (rollercoaster) compared to no VS for the different GVS intensities, both patients and HC performed with an increased sway speed for *no*GVS (PPPD: *t*(52) = 6.61, *p* < 0.001, *Δ* = 28.3, HC: *t*(52) = 8.11, *p* < 0.001, *Δ* = 36.02), *sham*GVS (PPPD: *t*(52) = 8.34, *p* < 0.001, *Δ* = 33.57, HC: *t*(52) = 9.09, *p* < 0.001, *Δ* = 36.02) and GVS (PPPD: *t*(52) = 5.89, *p* < 0.001, *Δ* = 43.02, HC: *t*(52) = 9.06, *p* < 0.001, *Δ* = 68.66). All post hoc comparisons are listed in the supplementary information (see Supplementary Table S7). Supplementary Figure SF1A emphasizes post hoc comparisons of PSS between PPPD and HC during VS without GVS on foam surface.

Regarding egomotion perception, there was a main effect for GROUP (*F*(1, 52) = 11.36, *p* = 0.001, *η*^2^ = 0.096), GVS (*F*(2, 104) = 369.16, *p* < 0.001, *η*^2^ = 0.518) and VS (*F*(3, 156) = 56.13, *p* < 0.001, *η*^2^ = 0.152). Interaction between GROUP and GVS was not significant (p = 0.674), neither was GROUP × VS (*p* = 0.75), the three-way interaction GROUP × GVS × VS showed a trend (*F*(6, 312) = 2.06, *p* = 0.057, *η*^2^ = 0.008, Fig. 5B). PPPD patients rated each GVS intensity higher than HC (*no*GVS: *t*(52) = 2.46, *p* = 0.017, *Δ* = 11.05; *sham*GVS: *t*(52) = 2.75, *p* = 0.008, *Δ* = 13.62; GVS: *t*(52) = 3.89, *p* < 0.001, *Δ* = 14.12). For VS, patients showed increased ratings for no VS (*t*(52) = 4.10, *p* < 0.001, *Δ* = 18.04), movie (*t*(52) = 2.86, *p* = 0.006, *Δ* = 10.65) and flow-field (*t*(52) = 3.27, *p* = 0.002, *Δ* = 13.94) but not for rollercoaster (*p* = 0.90). Evaluating the effect of complex VS (rollercoaster) on egomotion perception regarding the different GVS conditions, patients rated their egomotion perception during rollercoaster VS compared to no VS higher for *no*GVS (*t*(52) = 6.22, *p* < 0.001, *Δ* = 22.51), but not for *sham*GVS (*p* > *1.0*) nor GVS (*p* = *0.823*). In contrast, HC rated their egomotion perception during rollercoaster VS higher compared to the no VS condition for all GVS conditions (noGVS: *t*(52) = 6.36, *p* < 0.001, *Δ* = 23.89, *sham*GVS: *t*(52) = − 3.62, *p* = 0.004, *Δ* = 21.08, GVS: *t*(52) = 3.81, *p* = 0.002, *Δ* = 12.99). All post hoc comparisons are listed in the supplementary information (see Supplementary Table S8). Supplementary Figure SF1B emphasizes post hoc comparisons of egomotion perception between PPPD and HC during VS without GVS on foam.

REML-based variance decomposition revealed substantial interindividual variability, in particular in the responses to vestibular (GVS) and complex visual (rollercoaster) stimulation in PSS and egomotion perception (see Supplementary Table S9 and S10). This is in line with our ANOVA analysis highlighting GVS and the rollercoaster conditions with the largest impact on postural control. REML-based analysis not only disclosed that both manipulations reliably influence postural sway and egomotion perception but also that the magnitude of the responses differ markedly across participants, even after accounting for group differences between PPPD and HC.

### Correlation with clinical scores and questionnaires

**Fig. 6:**
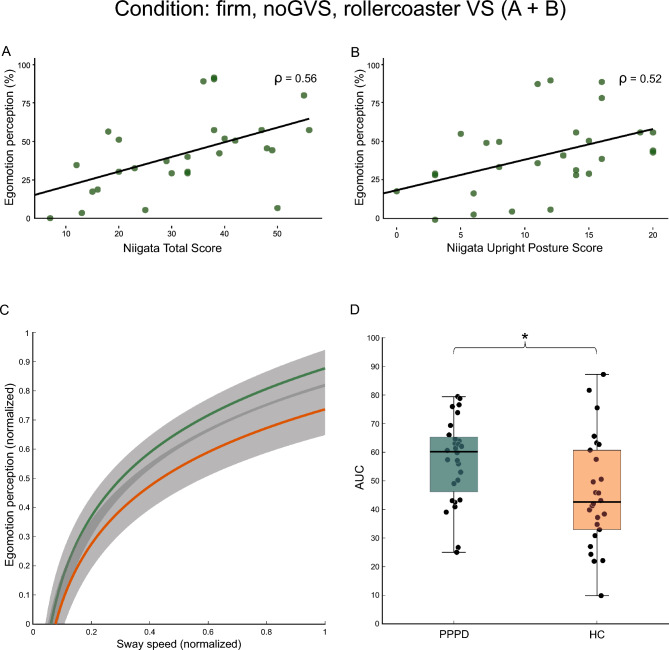
Correlation between PPPD patients’ egomotion perception with the NPQ total score (**A**) and the upright posture sub score (**B**) during *no*GVS and rollercoaster VS on firm surface. Patients with elevated NPQ scores revealed an increased level of egomotion perception that is also present when only considering the upright posture sub score. Normalized PSS and according egomotion perception for all conditions plotted for PPPD patients (green) and HC (orange) as a logarithmic fit (**C**) and their calculated area under the curve (**D**). PPPD patients presented increased egomotion perception during baseline PSS compared to HC that increased non-linearly with increasing PSS. Gray area: mean and 95% confidence interval. * ≤ 0.05. *AUC* area under the curve, *GVS* galvanic vestibular stimulation, *HC* healthy control subject, *NPQ* Niigata PPPD Questionnaire, *PPPD* persistent postural-perceptual dizziness, *PSS* postural sway speed, *VS* visual stimulation

We performed Spearman rho correlations between PSS and egomotion perception during the baseline condition (no VS + *no*GVS), the most demanding GVS condition (no VS + *GVS*), the most demanding VS condition (rollercoaster VS + *no*GVS), and their combination (*GVS* + rollercoaster VS) with clinical scores and questionnaires. Egomotion perception correlated with patients’ disease severity: patients with higher ratings of their perceived egomotion during rollercoaster VS and *no*GVS had an elevated score of the Niigata PPPD questionnaire (NPQ) (*p* = 0.007, *ρ* = 0.56, Fig. 6A). Dividing the NPQ into its sub-scores, the correlation was present for the movement sub score (*p* = 0.006, *ρ* = 0.574) as well as for the upright posture sub-score (*p* = 0.014, *ρ* = 0.52, Fig. 6B) but not for the visual sub-score (*p* = 0.109). For the upright posture sub-score, patients’ egomotion perception also correlated with increased scores during no VS and GVS (*p* = 0.020, *ρ* = 0.48) and rollercoaster VS and GVS (*p* = 0.012, *ρ* = 0.54). Neither the other sub-scores nor the total NPQ score correlated with patient’s egomotion perception during any other condition (*p* always > 0.081). There was no correlation between PSS and NPQ total nor sub-scores (*p* always > 0.625). We found no correlation between the ALQ (total nor sub-scores) and patients’ egomotion perception (*p* always > 0.06) nor PSS (*p* always > 0.325) during neither GVS nor VS. Lastly, we related individual PSS with egomotion perception for all conditions and plotted the normalized data as a logarithmic fit (Fig. 6C). The analysis of the AUC revealed distinctly different group dynamics: patients showed a significantly larger AUC (*t*(52) = 2.46, *p* = 0.017; Fig. 6D), i.e., a higher increase in individual egomotion perception with small PSS.

For correlations between patients’ PSS or egomotion perception with their disease duration, we excluded patients with a disease duration > 2 × SD (see Table 1) due to a wide range in the parameter disease duration (3–201 months). This resulted in the exclusion of one patient. There was no correlation regarding PSS (*p* = 0.409) nor egomotion perception (*p* = 0.813). There was no significant correlation between PSS or egomotion perception with the Hospital Anxiety and Depression Scale (HADS) (PPPD: *p* always > 0.317; HC: *p* always > 0.102) nor with the Motion Sickness Susceptibility Questionnaire (MSSQ; PPPD: *p* always > 0.862; HC: *p* always > 0.170).

## Discussion

Abnormal bottom-up central processing with alterations in motion perception, i.e., sensory-perceptual amplification [28], may influence top-down postural control and lead to postural misperception and maladaptation in PPPD [22, 25]. In our PPPD cohort, patients perceived variable degrees of egomotion during stance evoked by experimental visual, vestibular, and combined stimulation. They described the elicited egomotion as comparable to symptoms of dizziness and unsteadiness they encounter in their daily life. We compared perceived egomotion and postural sway during stance with and without vestibular and/or visual stimulation to investigate visual sensitivity and in particular how postural misperception is affected by motion cues.

Generally, PPPD patients reported a higher level of egomotion perception during all stimulus conditions (including conditions without sensory stimulation), in line with previous studies [9, 18, 28]. Vestibular stimulation (GVS) elicited a higher egomotion perception than visual stimulation (VS) in patients and HC.

### Effects of vestibular stimuli

In line with previous studies [9, 30], PSS of patients became higher compared to that of HC’s by GVS while standing on the firm surface. This abnormal sway disappeared when somatosensory signals were weakened (standing on foam), eliciting increased efforts on postural control.

Interestingly, not only PSS but also egomotion perception was higher during *sham*GVS compared to noGVS in both groups. Simply predicting an impending vestibular stimulus that evokes egomotion caused not only higher egomotion but also higher postural sway. Anxiety in PPPD may cause an abnormal prediction of egomotion during stance. However, the strong effect of the sham stimulus did not differ from that observed in HC, nor was there a significant correlation between anxiety scores and PSS or egomotion perception. This effect of the sham stimulus on egomotion perception is inherent to the upright body position (stance), as it was not previously observed in supine position [6, 28].

### Effects of visual motion and non-motion stimuli

Among all visual motion stimuli, rollercoaster stimulation elicited higher postural sway and egomotion perception in both groups, compared with the no visual motion condition (fixation). This is different from a related study in which PPPD patients showed an increased postural sway when facing another visual motion task, i.e., the Rod-and-Disk test [4]. Unfortunately, perceived egomotion was not recorded in the latter study. Despite significant differences in the magnitude of elicited egomotion, patients’ stimulus-related postural sway was not different from that of HC. Another study in which PPPD patients were exposed to moving stars through a head-mounted display showed indistinguishable postural sway between PPPD and HC [13]. This has been related to suspected muscle rigidity, possibly driven by anxiety. Our PPPD cohort had increased anxiety scores compared to HC but no significant correlation with PSS or egomotion perception. When depriving patients from somatosensory inputs (standing on foam), which leads to more anxiety, patients’ PSS and egomotion perception were increased compared to standing on firm surface. However, PSS and egomotion perception on foam was not different from that of HC. Finally, despite having motion components, the movie stimulus provoked the least egomotion perception and lowest sway. It seems to be egomotion rather than motion per se, that accounts for the patients’ dizziness.

### Effects of combined visual–vestibular stimuli

When applying vestibular stimulation (GVS) in combination with any VS, patients showed higher PSS compared to HC. This effect is largely driven by the vestibular stimulation, as it is not found in the noGVS condition. Vestibular stimulation seems to extinguish the strong effect by rollercoaster visual motion in PPPD but not in HC. The perceived egomotion was generally higher in patients but this increase was not related to the different visual motion stimuli, i.e., the influence of visual motion stimuli on egomotion perception is neglectable when concomitant vestibular stimulation is provided. Although the intensities of elicited egomotion by vestibular and visual motion stimuli are not matched (apparent by the higher ratings for GVS), the data suggest a stronger impact of vestibular compared to visual motion stimulation on postural sway and egomotion. While it was an experimental vestibular stimulus in our study (GVS), similar effects might be obtained when aversively reported visual motion stimuli are combined with active vestibular stimulation, i.e., head and body rotation during physiotherapy of PPPD patients.

As expected, postural sway became higher in the most challenging condition for postural control, i.e., standing on foam noticeably, however, without group differences. The difference in PSS between rollercoaster VS and the other VS conditions became stronger on foam compared to firm support. Complex (rollercoaster) visual motion stimuli powerfully destabilized posture when proprioceptive signals for postural control are weakened (foam), but this effect is indistinguishable in both groups and becomes even higher with additional non-predictive (sham) vestibular stimulation. This marked effect of rollercoaster visual motion on objective postural sway is not reflected by perceived egomotion. The higher egomotion perception during rollercoaster VS compared to the other VS fades away in patients with concomitant anticipated (*sham*GVS) or effective vestibular stimulation (GVS). In contrast, rollercoaster VS in HC maintains a stronger impact on egomotion perception compared to the other VS. Unlike PPPD, rollercoaster VS still profoundly increased egomotion perception of HC while standing on foam, implying that HC remain capable of discriminating visual stimuli. In contrast, the lack of further increase in egomotion perception of PPPD patients might be caused by a ceiling effect since egomotion ratings of patients were consistently higher than in HC and reached maximum values during GVS even without additional VS on foam. As this is not the case during noGVS or *sham*GVS, it argues against a particular visual sensitivity of postural control and egomotion perception in PPPD patients.

Movie VS elicited the least egomotion perception and PSS in both PPPD and HC, not only during VS alone but also in combination with GVS. PSS was even lower during movie VS alone compared to noVS. Movie VS possibly causes an allocation of attention toward the movie story which reduces egomotion perception and PSS. This is in line with other cognitive tasks in related studies (e.g., counting backwards, serial-3 subtraction task), in which patient’s sway became indistinguishable from HC [13] and improved when compared to baseline conditions (firm surface, noGVS) [30]. This cognitive strategy could potentially be used by physiotherapists and patients to reduce symptoms in their daily life.

Our statistical results were supported by the amendment of our maximum likelihood estimation analysis (REML) as it outlined the largest interindividual variability in response to vestibular (GVS) and complex visual (rollercoaster VS) stimulation, i.e., stimuli which elicited the largest level of egomotion perception. This indicates that individuals do not use a uniform sensory weighting strategy for visual and vestibular stimuli. Instead, they showed personalized patterns of visual and vestibular scaling. This is true both for the objectively measured postural sway as well as for the subjective perspective of egomotion perception.

### Postural misperception

Across all postural conditions, patients’ egomotion perception became higher with increasing PSS as reflected by their correlations. The relationship was non-linear, indicating a disproportionate fast increase in egomotion perception with increasing sway speed. This non-linear correlation was partially seen in HC, suggesting that perception of vestibular and visual motion stimuli is per se non-linear. However, the significant group difference in the area under the curve (AUC) may reflect a non-linear posturo-perceptual scaling process with faster ceiling effects as a crucial mechanism in PPPD.

The most significant difference between both groups is the higher baseline egomotion perception of patients. Any sensory (rollercoaster visual, GVS) stimulation increased postural sway and its rating in addition to this baseline. Our study does not provide evidence for postural misperception of PPPD patients during multi-sensory stimulation, i.e., sensory stimulation reduces abnormal sensory-perceptual scaling [28]. The data rather suggest an abnormal baseline neural activity contributing to continuous dizziness and perceived unsteadiness, in line with increased neural brain activity, possibly reflecting a neuronal amplification mechanism [28].

In line with the absence of a particular visual sensitivity of egomotion perception in our PPPD cohort, egomotion was not correlated with the visual intolerance PPPD subtype [1]. In contrast, egomotion perception increased strongly with the Niigata posture score and the PPPD subtype ‘*upright posture/quiet standing’*—particularly during rollercoaster VS—compared to the other subtypes.

## Limitations of the study

The different visual and vestibular stimuli were suitable and hence effective for our objective, as they were clearly distinguishable by both groups. However, GVS induced stronger PSS and egomotion perception compared to VS, indicating that the intensities of both stimulation approaches were not matched. Therefore, conclusion with respect to the dominance of any stimulation on PSS and egomotion is restricted.

## Conclusion

We demonstrated that visual stimuli with different levels of egomotion induction have a strong and differentiable effect on postural control and egomotion perception in PPPD patients. The combination of visual stimulation with vestibular stimulation overshadowed effects due to visual stimulation in PPPD patients, while healthy participants continued to show differentiable effects. PPPD patients consistently showed higher postural sway and egomotion perception as a baseline feature. Multisensory stimulation increased postural sway and egomotion perception but non-linearly, particularly in patients. This non-linear posturo-perceptual scaling process with faster ceiling effects distinguished both groups and points to a crucial mechanism in PPPD. In contrast, postural misperception seems to be a feature of PPPD patients during a quiet stance on a firm ground but can be reversed during multi-sensory stimulation, e.g., physiotherapy.

## Supplementary Information

Below is the link to the electronic supplementary material.

Supplementary file1 (MP4 70551 KB)

Supplementary file2 (DOCX 435 KB)

## Data Availability

Neurologic-clinical, questionnaire, and posturography data are not publicly available to preserve individuals’ privacy. The data are, however, available from the authors upon reasonable request.
