# Supplementary material for: Vestibular and visual influence on postural stability and egomotion perception in persistent postural-perceptual dizziness (PPPD)
Source: J Neurol. 2026 Feb 9;273(2):127. doi: 10.1007/s00415-026-13653-z (PMC12886305; doi:10.1007/s00415-026-13653-z)
Supplement: Supplementary file 2 — Supplementary file2 (DOCX 435 KB) [file 415_2026_13653_MOESM2_ESM.docx]

# Supplementary Information

# Methods:

### Video References:

Hazanavicius, M. (Director). (2011). The Artist [Film]. La Petite Reine**;** Studio 37**;** La Classe Américaine**;** JD Prod**;** France 3 Cinéma**;** Jouror Productions**;** uFilm**.**

4K AWESOME Twister Roller Coaster Front Seat POV Knoebels Amusement Park. Available (as of January, 2026): <https://www.youtube.com/watch?v=oAJLKDMihnU>

Tremors Front Row POV | Silverwood Theme Park 2022. Available (as of January, 2026): <https://www.youtube.com/watch?v=zgU8bQDpn-I>

Nitro front seat on-ride 4K POV @60fps Six Flags Great Adventure**.** Available (as of January, 2026)**:** <https://www.youtube.com/watch?v=ZmMLSUbRuoM>

# Results

## On firm surface

### Galvanic vestibular stimulation (GVS) on **firm surface**

**Table 1**: Pairwise comparisons of GVS per GROUP for PSS

| Contrast | Group | Estimate | SE | df | t.ratio | p.value |
| --- | --- | --- | --- | --- | --- | --- |
| noGVS- sham | PPPD | -8.8848 | 1.6997 | 52.00 | -5.2274 | **0.0000** |
| noGVS - GVS | PPPD | -37.8785 | 5.1650 | 52.00 | -7.3337 | **0.0000** |
| sham - GVS | PPPD | -28.9937 | 4.7111 | 52.00 | -6.1543 | **0.0000** |
| noGVS - sham | HC | -4.6393 | 1.7638 | 52.00 | -2.6303 | **0.0336** |
| noGVS - GVS | HC | -11.4570 | 5.3600 | 52.00 | -2.1375 | 0.1118 |
| sham - GVS | HC | -6.8178 | 4.8890 | 52.00 | -1.3945 | 0.5073 |

For better distinction between stimuli conditions are distinguished by grey color vs. unmarked fields. Abbreviations: df = degrees of freedom, HC = healthy control subjects, PPPD = persistent postural-perceptual dizziness, PSS = postural sway speed, SE = standard error, VS = visual stimulation.

**Table 2**: Pairwise comparisons of GVS per GROUP for egomotion perception

| Contrast | Group | Estimate | SE | df | t.ratio | p.value |
| --- | --- | --- | --- | --- | --- | --- |
| noGVS - Sham | PPPD | -24.3515 | 4.9290 | 52.00 | -4.9404 | **0.0000** |
| noGVS - GVS | PPPD | -58.5657 | 4.6232 | 52.00 | -12.6679 | **0.0000** |
| Sham - GVS | PPPD | -34.2143 | 4.4525 | 52.00 | -7.6843 | **0.0000** |
| noGVS - Sham | HC | -20.7901 | 5.1151 | 52.00 | -4.0645 | **0.0005** |
| noGVS - GVS | HC | -58.7911 | 4.7977 | 52.00 | -12.2541 | **0.0000** |
| Sham - GVS | HC | -38.0010 | 4.6206 | 52.00 | -8.2243 | **0.0000** |

For better distinction between stimuli conditions are distinguished by grey color vs. unmarked fields. Abbreviations: df = degrees of freedom, HC = healthy control subjects, PPPD = persistent postural-perceptual dizziness, PSS = postural sway speed, SE = standard error, VS = visual stimulation.

### Visual stimulation (VS) on **firm surface**

**Table 3**: Pairwise comparisons of VS per GROUP for PSS

| Contrast | Group | Estimate | SE | df | t-ratio | p-value |
| --- | --- | --- | --- | --- | --- | --- |
| No - RollerCoaster | PPPD | -5.8162 | 1.2752 | 52.00 | -4.5610 | **0.0002** |
| No - FlowField | PPPD | -1.1977 | 1.1557 | 52.00 | -1.0363 | 1.0000 |
| No - Movie | PPPD | -1.5617 | 0.9728 | 52.00 | -1.6053 | 0.6869 |
| RollerCoaster - FlowField | PPPD | 4.6185 | 1.0824 | 52.00 | 4.2669 | **0.0005** |
| RollerCoaster - Movie | PPPD | 4.2545 | 1.3808 | 52.00 | 3.0812 | **0.0198** |
| FlowField - Movie | PPPD | -0.3640 | 0.8710 | 52.00 | -0.4179 | 1.0000 |
| No - RollerCoaster | HC | -5.6975 | 1.3234 | 52.00 | -4.3053 | **0.0004** |
| No - FlowField | HC | -3.1114 | 1.1993 | 52.00 | -2.5943 | 0.0737 |
| No - Movie | HC | -2.2401 | 1.0096 | 52.00 | -2.2189 | 0.1853 |
| RollerCoaster - FlowField | HC | 2.5861 | 1.1233 | 52.00 | 2.3023 | 0.1521 |
| RollerCoaster - Movie | HC | 3.4574 | 1.4330 | 52.00 | 2.4128 | 0.1164 |
| FlowField - Movie | HC | 0.8713 | 0.9039 | 52.00 | 0.9640 | 1.0000 |

For better distinction between stimuli conditions are distinguished by grey color vs. unmarked fields. Abbreviations: df = degrees of freedom, HC = healthy control subjects, PPPD = persistent postural-perceptual dizziness, PSS = postural sway speed, SE = standard error, VS = visual stimulation.

**Table 4**: Pairwise comparisons of VS per GROUP for egomotion perception

| Contrast | Group | Estimate | SE | df | t.ratio | p.value |
| --- | --- | --- | --- | --- | --- | --- |
| No - RollerCoaster | PPPD | -18.8163 | 4.8635 | 52.00 | -3.8689 | **0.0018** |
| No - FlowField | PPPD | 4.6541 | 3.6034 | 52.00 | 1.2916 | 1.0000 |
| No - Movie | PPPD | 10.8234 | 3.3677 | 52.00 | 3.2139 | **0.0135** |
| RollerCoaster - FlowField | PPPD | 23.4704 | 4.4023 | 52.00 | 5.3314 | **0.0000** |
| RollerCoaster - Movie | PPPD | 29.6397 | 4.0782 | 52.00 | 7.2679 | **0.0000** |
| FlowField - Movie | PPPD | 6.1692 | 2.0533 | 52.00 | 3.0046 | **0.0245** |
| No - RollerCoaster | HC | -9.9043 | 5.0471 | 52.00 | -1.9624 | 0.3305 |
| No - FlowField | HC | 0.8302 | 3.7394 | 52.00 | 0.2220 | 1.0000 |
| No - Movie | HC | 1.9098 | 3.4948 | 52.00 | 0.5465 | 1.0000 |
| RollerCoaster - FlowField | HC | 10.7345 | 4.5685 | 52.00 | 2.3497 | 0.1357 |
| RollerCoaster - Movie | HC | 11.8141 | 4.2321 | 52.00 | 2.7915 | **0.0439** |
| FlowField - Movie | HC | 1.0796 | 2.1308 | 52.00 | 0.5067 | 1.0000 |

For better distinction between stimuli conditions are distinguished by grey color vs. unmarked fields. Abbreviations: df = degrees of freedom, HC = healthy control subjects, PPPD = persistent postural-perceptual dizziness, PSS = postural sway speed, SE = standard error, VS = visual stimulation.

### Visual and galvanic vestibular stimulation (VS + GVS) on **firm surface**

**Table 5**: Pairwise comparisons of VS per GVS and GROUP for PSS

| contrast | GVS | Gruppe | estimate | SE | df | t.ratio | p.value |
| --- | --- | --- | --- | --- | --- | --- | --- |
| No - RollerCoaster | noGVS | PPPD | -5.8162 | 1.2752 | 52.00 | -4.5610 | **0.0002** |
| No - FlowField | noGVS | PPPD | -1.1977 | 1.1557 | 52.00 | -1.0363 | 1.0000 |
| No - Movie | noGVS | PPPD | -1.5617 | 0.9728 | 52.00 | -1.6053 | 0.6869 |
| RollerCoaster  - FlowField | noGVS | PPPD | 4.6185 | 1.0824 | 52.00 | 4.2669 | **0.0005** |
| RollerCoaster - Movie | noGVS | PPPD | 4.2545 | 1.3808 | 52.00 | 3.0812 | **0.0198** |
| FlowField - Movie | noGVS | PPPD | -0.3640 | 0.8710 | 52.00 | -0.4179 | 1.0000 |
| No - RollerCoaster | Sham | PPPD | -15.1280 | 4.8113 | 52.00 | -3.1443 | **0.0165** |
| No - FlowField | Sham | PPPD | 4.2828 | 1.4503 | 52.00 | 2.9531 | **0.0283** |
| No - Movie | Sham | PPPD | 6.6224 | 1.5711 | 52.00 | 4.2152 | **0.0006** |
| RollerCoaster  - FlowField | Sham | PPPD | 19.4108 | 5.2381 | 52.00 | 3.7057 | **0.0031** |
| RollerCoaster - Movie | Sham | PPPD | 21.7504 | 5.4456 | 52.00 | 3.9941 | **0.0012** |
| FlowField - Movie | Sham | PPPD | 2.3396 | 1.2129 | 52.00 | 1.9289 | 0.3553 |
| No - RollerCoaster | GVS | PPPD | -14.1771 | 7.7574 | 52.00 | -1.8276 | 0.4401 |
| No - FlowField | GVS | PPPD | 0.8150 | 4.2468 | 52.00 | 0.1919 | 1.0000 |
| No - Movie | GVS | PPPD | 17.8382 | 5.4957 | 52.00 | 3.2458 | **0.0123** |
| RollerCoaster  - FlowField | GVS | PPPD | 14.9921 | 7.9091 | 52.00 | 1.8956 | 0.3815 |
| RollerCoaster - Movie | GVS | PPPD | 32.0154 | 8.1738 | 52.00 | 3.9168 | **0.0016** |
| FlowField - Movie | GVS | PPPD | 17.0233 | 4.8045 | 52.00 | 3.5432 | **0.0051** |
| No - RollerCoaster | noGVS | HC | -5.6975 | 1.3234 | 52.00 | -4.3053 | **0.0004** |
| No - FlowField | noGVS | HC | -3.1114 | 1.1993 | 52.00 | -2.5943 | 0.0737 |
| No - Movie | noGVS | HC | -2.2401 | 1.0096 | 52.00 | -2.2189 | 0.1853 |
| RollerCoaster  - FlowField | noGVS | HC | 2.5861 | 1.1233 | 52.00 | 2.3023 | 0.1521 |
| RollerCoaster - Movie | noGVS | HC | 3.4574 | 1.4330 | 52.00 | 2.4128 | 0.1164 |
| FlowField - Movie | noGVS | HC | 0.8713 | 0.9039 | 52.00 | 0.9640 | 1.0000 |
| No - RollerCoaster | Sham | HC | -12.8712 | 4.9929 | 52.00 | -2.5779 | 0.0769 |
| No - FlowField | Sham | HC | 2.4920 | 1.5050 | 52.00 | 1.6558 | 0.6227 |
| No - Movie | Sham | HC | 4.0816 | 1.6304 | 52.00 | 2.5035 | 0.0929 |
| RollerCoaster  - FlowField | Sham | HC | 15.3632 | 5.4358 | 52.00 | 2.8263 | **0.0400** |
| RollerCoaster - Movie | Sham | HC | 16.9528 | 5.6512 | 52.00 | 2.9999 | **0.0248** |
| FlowField - Movie | Sham | HC | 1.5896 | 1.2587 | 52.00 | 1.2628 | 1.0000 |
| No - RollerCoaster | GVS | HC | -29.7049 | 8.0503 | 52.00 | -3.6899 | **0.0032** |
| No - FlowField | GVS | HC | -2.0852 | 4.4071 | 52.00 | -0.4732 | 1.0000 |
| No - Movie | GVS | HC | 3.7531 | 5.7032 | 52.00 | 0.6581 | 1.0000 |
| RollerCoaster  - FlowField | GVS | HC | 27.6197 | 8.2077 | 52.00 | 3.3651 | **0.0087** |
| RollerCoaster - Movie | GVS | HC | 33.4580 | 8.4824 | 52.00 | 3.9444 | **0.0014** |
| FlowField - Movie | GVS | HC | 5.8383 | 4.9859 | 52.00 | 1.1710 | 1.0000 |

For better distinction between stimuli conditions are distinguished by grey color vs. unmarked fields. Abbreviations: df = degrees of freedom, GVS = galvanic vestibular stimulation, HC = healthy control subjects, PPPD = persistent postural-perceptual dizziness, PSS = postural sway speed, SE = standard error, VS = visual stimulation.

**Table 6**: Pairwise comparisons of VS per GVS and GROUP for egomotion perception

| contrast | GVS | Gruppe | estimate | SE | df | t.ratio | p.value |
| --- | --- | --- | --- | --- | --- | --- | --- |
| No - RollerCoaster | noGVS | PPPD | -18.8163 | 4.8635 | 52.00 | -3.8689 | **0.0018** |
| No - FlowField | noGVS | PPPD | 4.6541 | 3.6034 | 52.00 | 1.2916 | 1.0000 |
| No - Movie | noGVS | PPPD | 10.8234 | 3.3677 | 52.00 | 3.2139 | **0.0135** |
| RollerCoaster  - FlowField | noGVS | PPPD | 23.4704 | 4.4023 | 52.00 | 5.3314 | **0.0000** |
| RollerCoaster - Movie | noGVS | PPPD | 29.6397 | 4.0782 | 52.00 | 7.2679 | **0.0000** |
| FlowField - Movie | noGVS | PPPD | 6.1692 | 2.0533 | 52.00 | 3.0046 | **0.0245** |
| No - RollerCoaster | Sham | PPPD | 0.2916 | 4.8723 | 52.00 | 0.0599 | 1.0000 |
| No - FlowField | Sham | PPPD | 12.2698 | 3.6875 | 52.00 | 3.3274 | **0.0097** |
| No - Movie | Sham | PPPD | 18.9500 | 4.2154 | 52.00 | 4.4954 | **0.0002** |
| RollerCoaster  - FlowField | Sham | PPPD | 11.9781 | 4.3454 | 52.00 | 2.7565 | **0.0482** |
| RollerCoaster - Movie | Sham | PPPD | 18.6584 | 4.9325 | 52.00 | 3.7828 | **0.0024** |
| FlowField - Movie | Sham | PPPD | 6.6802 | 2.8268 | 52.00 | 2.3632 | 0.1314 |
| No - RollerCoaster | GVS | PPPD | 0.5640 | 2.4490 | 52.00 | 0.2303 | 1.0000 |
| No - FlowField | GVS | PPPD | 6.2406 | 3.4534 | 52.00 | 1.8071 | 0.4592 |
| No - Movie | GVS | PPPD | 10.6053 | 3.3870 | 52.00 | 3.1312 | **0.0171** |
| RollerCoaster  - FlowField | GVS | PPPD | 5.6766 | 3.8490 | 52.00 | 1.4748 | 0.8777 |
| RollerCoaster - Movie | GVS | PPPD | 10.0413 | 3.7655 | 52.00 | 2.6667 | **0.0611** |
| FlowField - Movie | GVS | PPPD | 4.3647 | 2.8601 | 52.00 | 1.5261 | 0.7983 |
| No - RollerCoaster | noGVS | HC | -9.9043 | 5.0471 | 52.00 | -1.9624 | 0.3305 |
| No - FlowField | noGVS | HC | 0.8302 | 3.7394 | 52.00 | 0.2220 | 1.0000 |
| No - Movie | noGVS | HC | 1.9098 | 3.4948 | 52.00 | 0.5465 | 1.0000 |
| RollerCoaster  - FlowField | noGVS | HC | 10.7345 | 4.5685 | 52.00 | 2.3497 | 0.1357 |
| RollerCoaster - Movie | noGVS | HC | 11.8141 | 4.2321 | 52.00 | 2.7915 | **0.0439** |
| FlowField - Movie | noGVS | HC | 1.0796 | 2.1308 | 52.00 | 0.5067 | 1.0000 |
| No - RollerCoaster | Sham | HC | 0.8966 | 5.0563 | 52.00 | 0.1773 | 1.0000 |
| No - FlowField | Sham | HC | 9.7504 | 3.8267 | 52.00 | 2.5480 | 0.0830 |
| No - Movie | Sham | HC | 15.1847 | 4.3745 | 52.00 | 3.4712 | **0.0063** |
| RollerCoaster  - FlowField | Sham | HC | 8.8538 | 4.5094 | 52.00 | 1.9634 | 0.3297 |
| RollerCoaster - Movie | Sham | HC | 14.2882 | 5.1187 | 52.00 | 2.7914 | **0.0439** |
| FlowField - Movie | Sham | HC | 5.4344 | 2.9335 | 52.00 | 1.8525 | 0.4178 |
| No - RollerCoaster | GVS | HC | 2.3747 | 2.5414 | 52.00 | 0.9344 | 1.0000 |
| No - FlowField | GVS | HC | 6.9837 | 3.5838 | 52.00 | 1.9487 | 0.3404 |
| No - Movie | GVS | HC | 9.2505 | 3.5149 | 52.00 | 2.6318 | 0.0669 |
| RollerCoaster  - FlowField | GVS | HC | 4.6090 | 3.9943 | 52.00 | 1.1539 | 1.0000 |
| RollerCoaster - Movie | GVS | HC | 6.8758 | 3.9076 | 52.00 | 1.7596 | 0.5062 |
| FlowField - Movie | GVS | HC | 2.2668 | 2.9680 | 52.00 | 0.7637 | 1.0000 |

For better distinction between stimuli conditions are distinguished by grey color vs. unmarked fields. Abbreviations: df = degrees of freedom, GVS = galvanic vestibular stimulation, HC = healthy control subjects, PPPD = persistent postural-perceptual dizziness, PSS = postural sway speed, SE = standard error, VS = visual stimulation.

## On foam surface

**A**


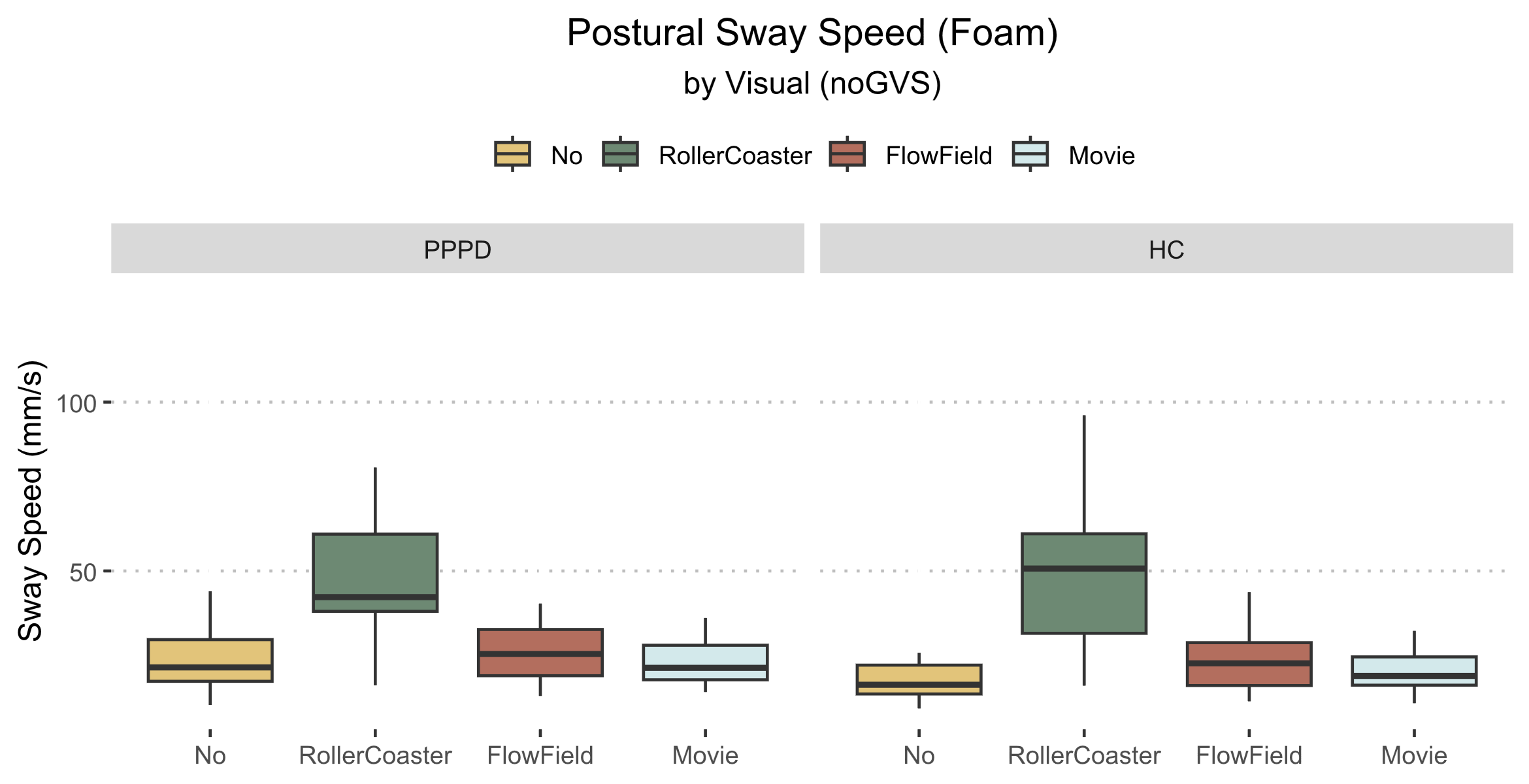


**B**


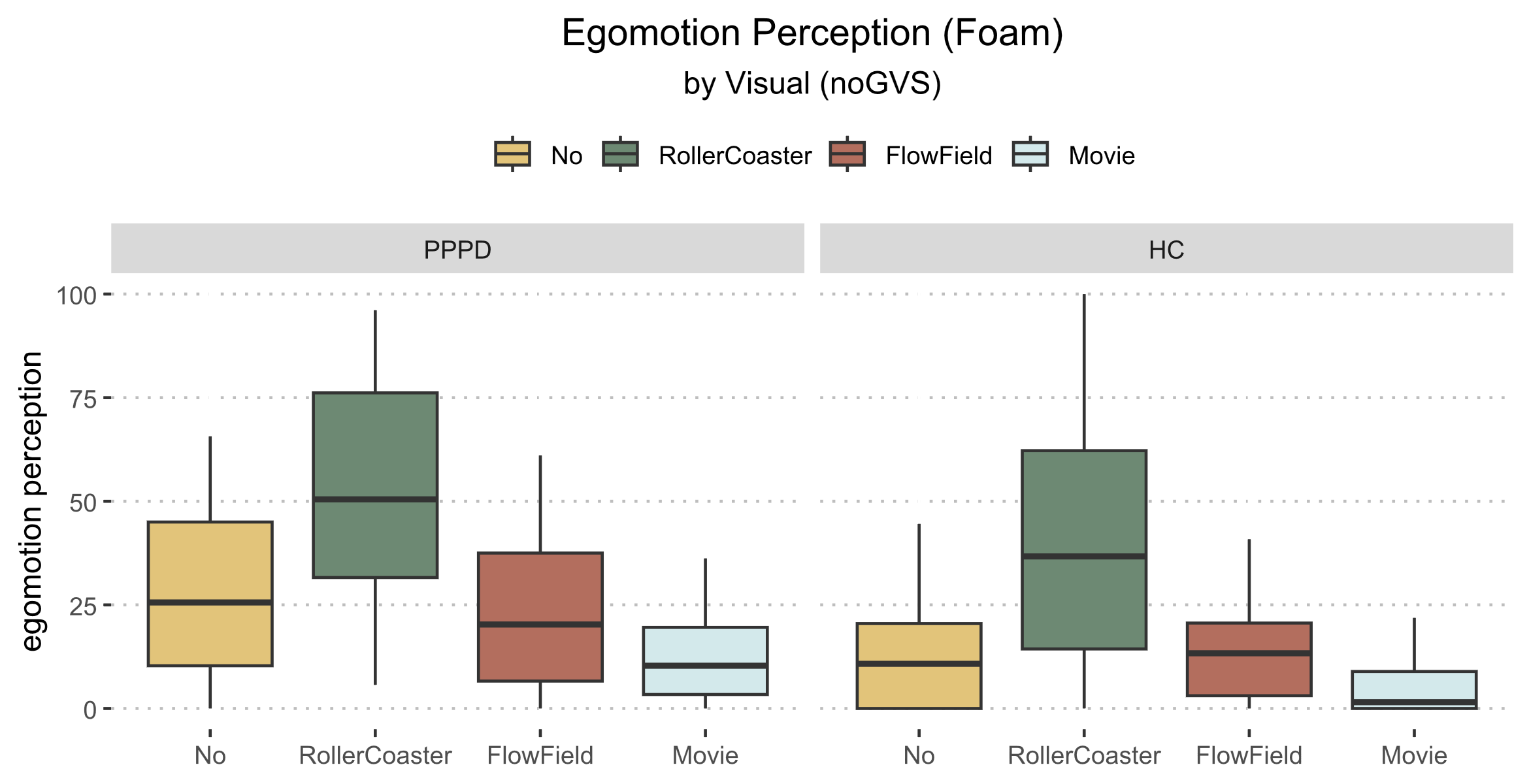


**Supplementary Fig.1**: PSS (**A**) and egomotion perception (**B**) for no, movie, flowfield and rollercoaster VS without GVS in PPPD patients and HC on foam surface. During any VS, PPPD patients and HC showed no group difference. Comparing results to firm surface, rollercoaster VS distinctly increases PSS and egomotion perception, particularly in HC. Abbreviations: HC = healthy control subjects, PPPD = persistent postural-perceptual dizziness, PSS = postural sway speed, VS = visual stimulation.

### Visual and galvanic vestibular stimulation (VS + GVS) on **foam surface**

**Table 7**: Pairwise comparisons of VS per GVS and GROUP for PSS

| contrast | GVS | Gruppe | estimate | SE | df | t.ratio | p.value |
| --- | --- | --- | --- | --- | --- | --- | --- |
| No - RollerCoaster | noGVS | PPPD | -28.2935 | 4.2788 | 52.00 | -6.6125 | **0.0000** |
| No - FlowField | noGVS | PPPD | -2.4043 | 1.6641 | 52.00 | -1.4448 | 0.9272 |
| No - Movie | noGVS | PPPD | 0.3276 | 1.3602 | 52.00 | 0.2409 | 1.0000 |
| RollerCoaster  - FlowField | noGVS | PPPD | 25.8892 | 4.3312 | 52.00 | 5.9774 | **0.0000** |
| RollerCoaster  - Movie | noGVS | PPPD | 28.6211 | 4.2857 | 52.00 | 6.6783 | **0.0000** |
| FlowField - Movie | noGVS | PPPD | 2.7319 | 1.2150 | 52.00 | 2.2484 | 0.1729 |
| No - RollerCoaster | Sham | PPPD | -33.5741 | 4.0260 | 52.00 | -8.3393 | **0.0000** |
| No - FlowField | Sham | PPPD | -9.7361 | 2.1282 | 52.00 | -4.5748 | **0.0002** |
| No - Movie | Sham | PPPD | -10.2496 | 2.0240 | 52.00 | -5.0640 | **0.0000** |
| RollerCoaster  - FlowField | Sham | PPPD | 23.8379 | 3.0960 | 52.00 | 7.6995 | **0.0000** |
| RollerCoaster  - Movie | Sham | PPPD | 23.3245 | 3.6286 | 52.00 | 6.4279 | **0.0000** |
| FlowField - Movie | Sham | PPPD | -0.5135 | 1.7580 | 52.00 | -0.2921 | 1.0000 |
| No - RollerCoaster | GVS | PPPD | -43.0191 | 7.3007 | 52.00 | -5.8924 | **0.0000** |
| No - FlowField | GVS | PPPD | 1.9499 | 4.0263 | 52.00 | 0.4843 | 1.0000 |
| No - Movie | GVS | PPPD | -4.6541 | 4.3513 | 52.00 | -1.0696 | 1.0000 |
| RollerCoaster  - FlowField | GVS | PPPD | 44.9690 | 6.9619 | 52.00 | 6.4593 | **0.0000** |
| RollerCoaster  - Movie | GVS | PPPD | 38.3650 | 5.9543 | 52.00 | 6.4432 | **0.0000** |
| FlowField - Movie | GVS | PPPD | -6.6040 | 3.3866 | 52.00 | -1.9501 | 0.3394 |
| No - RollerCoaster | noGVS | HC | -36.0214 | 4.4403 | 52.00 | -8.1124 | **0.0000** |
| No - FlowField | noGVS | HC | -6.4832 | 1.7270 | 52.00 | -3.7541 | **0.0026** |
| No - Movie | noGVS | HC | -2.6147 | 1.4116 | 52.00 | -1.8523 | 0.4180 |
| RollerCoaster  - FlowField | noGVS | HC | 29.5382 | 4.4947 | 52.00 | 6.5718 | **0.0000** |
| RollerCoaster  - Movie | noGVS | HC | 33.4068 | 4.4475 | 52.00 | 7.5114 | **0.0000** |
| FlowField - Movie | noGVS | HC | 3.8685 | 1.2609 | 52.00 | 3.0680 | **0.0205** |
| No - RollerCoaster | Sham | HC | -37.9907 | 4.1780 | 52.00 | -9.0931 | **0.0000** |
| No - FlowField | Sham | HC | -11.7116 | 2.2085 | 52.00 | -5.3029 | **0.0000** |
| No - Movie | Sham | HC | -13.2072 | 2.1004 | 52.00 | -6.2878 | **0.0000** |
| RollerCoaster  - FlowField | Sham | HC | 26.2792 | 3.2129 | 52.00 | 8.1792 | **0.0000** |
| RollerCoaster  - Movie | Sham | HC | 24.7835 | 3.7656 | 52.00 | 6.5816 | **0.0000** |
| FlowField - Movie | Sham | HC | -1.4956 | 1.8244 | 52.00 | -0.8198 | 1.0000 |
| No - RollerCoaster | GVS | HC | -68.6658 | 7.5763 | 52.00 | -9.0632 | **0.0000** |
| No - FlowField | GVS | HC | -4.8552 | 4.1783 | 52.00 | -1.1620 | 1.0000 |
| No - Movie | GVS | HC | -23.2962 | 4.5156 | 52.00 | -5.1590 | **0.0000** |
| RollerCoaster  - FlowField | GVS | HC | 63.8107 | 7.2247 | 52.00 | 8.8323 | **0.0000** |
| RollerCoaster  - Movie | GVS | HC | 45.3697 | 6.1791 | 52.00 | 7.3424 | **0.0000** |
| FlowField - Movie | GVS | HC | -18.4410 | 3.5144 | 52.00 | -5.2473 | **0.0000** |

For better distinction between stimuli conditions are distinguished by grey color vs. unmarked fields. Abbreviations: df = degrees of freedom, GVS = galvanic vestibular stimulation, HC = healthy control subjects, PPPD = persistent postural-perceptual dizziness, PSS = postural sway speed, SE = standard error, VS = visual stimulation.

**Table 8**: Pairwise comparisons of VS per GVS and GROUP for egomotion perception

| contrast | GVS | Gruppe | estimate | SE | df | t.ratio | p.value |
| --- | --- | --- | --- | --- | --- | --- | --- |
| No - RollerCoaster | noGVS | PPPD | -22.5142 | 3.6207 | 52.00 | -6.2182 | **0.0000** |
| No - FlowField | noGVS | PPPD | 4.8362 | 3.4593 | 52.00 | 1.3980 | 1.0000 |
| No - Movie | noGVS | PPPD | 14.6171 | 3.0836 | 52.00 | 4.7402 | **0.0001** |
| RollerCoaster  - FlowField | noGVS | PPPD | 27.3503 | 4.2032 | 52.00 | 6.5070 | **0.0000** |
| RollerCoaster  - Movie | noGVS | PPPD | 37.1313 | 4.4677 | 52.00 | 8.3110 | **0.0000** |
| FlowField - Movie | noGVS | PPPD | 9.7809 | 2.3268 | 52.00 | 4.2037 | **0.0006** |
| No - RollerCoaster | Sham | PPPD | -3.6483 | 5.6128 | 52.00 | -0.6500 | 1.0000 |
| No - FlowField | Sham | PPPD | 12.2896 | 3.9852 | 52.00 | 3.0838 | **0.0196** |
| No - Movie | Sham | PPPD | 13.0569 | 4.4931 | 52.00 | 2.9060 | **0.0322** |
| RollerCoaster  - FlowField | Sham | PPPD | 15.9379 | 4.0947 | 52.00 | 3.8923 | **0.0017** |
| RollerCoaster  - Movie | Sham | PPPD | 16.7053 | 3.8210 | 52.00 | 4.3720 | **0.0004** |
| FlowField - Movie | Sham | PPPD | 0.7673 | 3.2809 | 52.00 | 0.2339 | 1.0000 |
| No - RollerCoaster | GVS | PPPD | -4.9615 | 3.2864 | 52.00 | -1.5097 | 0.8230 |
| No - FlowField | GVS | PPPD | 3.7517 | 3.6489 | 52.00 | 1.0282 | 1.0000 |
| No - Movie | GVS | PPPD | 2.4208 | 3.7198 | 52.00 | 0.6508 | 1.0000 |
| RollerCoaster  - FlowField | GVS | PPPD | 8.7133 | 3.5668 | 52.00 | 2.4429 | 0.1080 |
| RollerCoaster  - Movie | GVS | PPPD | 7.3823 | 2.8453 | 52.00 | 2.5946 | 0.0737 |
| FlowField - Movie | GVS | PPPD | -1.3309 | 2.5478 | 52.00 | -0.5224 | 1.0000 |
| No - RollerCoaster | noGVS | HC | -23.8921 | 3.7573 | 52.00 | -6.3588 | **0.0000** |
| No - FlowField | noGVS | HC | -0.4931 | 3.5899 | 52.00 | -0.1374 | 1.0000 |
| No - Movie | noGVS | HC | 8.8695 | 3.2000 | 52.00 | 2.7717 | **0.0463** |
| RollerCoaster  - FlowField | noGVS | HC | 23.3990 | 4.3619 | 52.00 | 5.3644 | **0.0000** |
| RollerCoaster  - Movie | noGVS | HC | 32.7616 | 4.6364 | 52.00 | 7.0662 | **0.0000** |
| FlowField - Movie | noGVS | HC | 9.3626 | 2.4146 | 52.00 | 3.8775 | **0.0018** |
| No - RollerCoaster | Sham | HC | -21.0808 | 5.8247 | 52.00 | -3.6192 | **0.0040** |
| No - FlowField | Sham | HC | 0.7072 | 4.1357 | 52.00 | 0.1710 | 1.0000 |
| No - Movie | Sham | HC | -2.2444 | 4.6627 | 52.00 | -0.4813 | 1.0000 |
| RollerCoaster  - FlowField | Sham | HC | 21.7879 | 4.2493 | 52.00 | 5.1275 | **0.0000** |
| RollerCoaster  - Movie | Sham | HC | 18.8364 | 3.9652 | 52.00 | 4.7504 | **0.0001** |
| FlowField - Movie | Sham | HC | -2.9515 | 3.4047 | 52.00 | -0.8669 | 1.0000 |
| No - RollerCoaster | GVS | HC | -12.9882 | 3.4105 | 52.00 | -3.8083 | **0.0022** |
| No - FlowField | GVS | HC | 8.3633 | 3.7867 | 52.00 | 2.2086 | 0.1898 |
| No - Movie | GVS | HC | 1.3088 | 3.8602 | 52.00 | 0.3390 | 1.0000 |
| RollerCoaster  - FlowField | GVS | HC | 21.3514 | 3.7014 | 52.00 | 5.7685 | **0.0000** |
| RollerCoaster  - Movie | GVS | HC | 14.2970 | 2.9527 | 52.00 | 4.8420 | **0.0001** |
| FlowField - Movie | GVS | HC | -7.0545 | 2.6440 | 52.00 | -2.6681 | 0.0609 |

For better distinction between stimuli conditions are distinguished by grey color vs. unmarked fields. Abbreviations: df = degrees of freedom, GVS = galvanic vestibular stimulation, HC = healthy control subjects, PPPD = persistent postural-perceptual dizziness, PSS = postural sway speed, SE = standard error, VS = visual stimulation.

**Table 9:** Restricted-maximum-likelihood-based variance decomposition with sway speed as the dependent variable

| **Dependent Variable** | **Random Effect** | **Effect** | **Std. Deviation** |
| --- | --- | --- | --- |
| Sway Speed | Subject | (Intercept) | 0.000000 |
| Sway Speed | Subject | GVSSham | 5.715992 |
| Sway Speed | Subject | GVS-GVS | 19.659533 |
| Sway Speed | Subject | VisualRollerCoaster | 17.294452 |
| Sway Speed | Subject | VisualFlowField | 2.551194 |
| Sway Speed | Subject | VisualMovie | 5.142127 |
| Sway Speed | Residual |  | 12.499816 |

Abbreviation: std. = standard.

**Table 10:** Restricted-maximum-likelihood-based variance decomposition with egomotion perception as the dependent variable

| **Dependent Variable** | **Random Effect** | **Effect** | **Std. Deviation** |
| --- | --- | --- | --- |
| Egomotion Perception | Subject | (Intercept) | 10.233569 |
| Egomotion Perception | Subject | GVSSham | 7.592207 |
| Egomotion Perception | Subject | GVS-GVS | 13.553300 |
| Egomotion Perception | Subject | VisualRollerCoaster | 10.608564 |
| Egomotion Perception | Subject | VisualFlowField | 6.200938 |
| Egomotion Perception | Subject | VisualMovie | 7.239838 |
| Egomotion Perception | Residual |  | 12.307065 |

Abbreviation: std. = standard.
